# Epigenetic Rewiring of Enhancer–Promoter Interactions in Cancer: Mechanisms and Therapeutic Implications

**DOI:** 10.3390/cancers18213416

**Published:** 2026-10-22

**Authors:** Lavanya Choppavarapu, Bakhtiyor Nosirov, Michael Kahn, Victor X. Jin

**Affiliations:** 1 Data Science Institute and MCW Cancer Center, Medical College of Wisconsin, Milwaukee, WI 53226, USA; choppavarapu@mcw.edu (L.C.); bnosirov@mcw.edu (B.N.); 2 Department of Cancer Biology and Molecular Medicine, Beckman Research Institute, City of Hope, Duarte, CA 91010, USA; mkahn@coh.org

**Keywords:** epigenetic rewiring, enhancer–promoter interactions, cancer

## Abstract

Cancer is increasingly recognized as a disease of transcriptional dysregulation in which genetic alterations and epigenetic remodeling cooperate to reprogram gene regulatory networks that govern cellular identity, proliferation, differentiation, and therapeutic response. Enhancer–promoter interactions are an important component of the regulatory systems that establish cell-type-specific gene expression and lineage identity. Cancer cells exploit the intrinsic plasticity of the epigenome to remodel enhancer landscapes, redistribute lineage-specific transcription factors (TFs), alter chromatin accessibility, reorganize three-dimensional (3D) genome architecture, and establish novel enhancer–promoter interactions that sustain oncogenic transcriptional programs. In this review, we discuss how enhancer–promoter communication is established and maintained within the 3D genome and examine the molecular mechanisms and drivers underlying epigenetic rewiring of enhancer–promoter interactions in cancer. We also highlight how the alterations of these epigenetic processes contribute to cancer initiation, progression, metastasis, and therapeutic resistance. Finally, we discuss epigenetic therapeutic strategies and emerging genomic technologies that exploit aberrant enhancer–promoter interactions to advance precision oncology.

## Simple Summary

Cancer cells can change the way genes are regulated by altering the communication between enhancers and promoters, which are DNA regions that help control gene activity. These interactions are influenced by epigenetic mechanisms and the three-dimensional organization of DNA within the cell nucleus. When these regulatory relationships are disrupted, cancer cells may activate genes that promote tumor growth, progression, metastasis, and resistance to treatment. Such changes can occur either through epigenetic remodeling without alterations in DNA sequences or through genetic changes, including mutations and chromosomal rearrangements, that create new regulatory connections. This review examines how enhancer–promoter interactions are altered in cancer, how current genomic technologies can help us to investigate these changes, and why evidence of enhancer activity or physical contact alone does not necessarily establish a functional regulatory relationship. We also discuss emerging therapeutic strategies targeting abnormal gene-regulatory networks. Understanding these mechanisms may help us to identify new biomarkers and therapeutic opportunities for improving cancer diagnosis and treatment.

## 1. Introduction

Cancer is increasingly recognized as a disease of transcriptional dysregulation, in which genetic alterations and epigenetic remodeling cooperate to reprogram gene regulatory networks that govern cellular identity, proliferation, differentiation, and therapeutic response [1,2,3]. Epigenetic mechanisms, including DNA methylation, histone modifications, ATP-dependent chromatin remodeling, and higher-order three-dimensional (3D) genome organization, collectively determine when, where, and to what extent genes are expressed [2,4,5]. Unlike genetic mutations, epigenetic alterations are highly plastic and reversible, enabling cancer cells to rapidly adapt to environmental cues, metabolic stress, immune surveillance, and therapeutic pressure [3,6]. Consequently, epigenetic plasticity has emerged as a defining feature of tumor evolution, intratumoral heterogeneity, metastatic dissemination, and treatment resistance [1,2,3,4].

Enhancer–promoter interactions are an important component of the regulatory systems that establish cell-type-specific gene expression and lineage identity [7,8,9]. Enhancers and promoters communicate through highly coordinated interactions mediated by transcription factors (TFs), coactivator complexes such as Mediator and *CBP*/*p300*, architectural proteins including *CTCF* and cohesin, and enhancer-derived RNAs (eRNAs). These interactions establish precise spatiotemporal transcriptional programs required for normal development, tissue homeostasis, and cellular differentiation [7,8,9,10]. The ability of distal enhancers to regulate their cognate target genes depends on the hierarchical organization of the genome within the 3D nuclear space. Recent advances in chromosome conformation capture technologies, including Hi-C, Micro-C, Capture-C, HiChIP, ChIA-PET, and single-cell chromatin conformation profiling [5,11,12,13], have fundamentally transformed our understanding of genome organization, demonstrating that enhancer–promoter interactions are dynamic, probabilistic, and highly context dependent.

An emerging paradigm in cancer biology is that malignant transformation frequently results from the epigenetic rewiring of enhancer–promoter interactions. Cancer cells exploit the intrinsic plasticity of the epigenome to remodel enhancer landscapes, redistribute lineage-specific transcription factors, alter chromatin accessibility, reorganize 3D genome architecture, and establish novel enhancer–promoter interactions that sustain oncogenic transcriptional programs [6,7,10,14,15]. For example, in acute myeloid leukemia (AML) and T-cell acute lymphoblastic leukemia (T-ALL), aberrant enhancer activation and chromatin looping are implicated in persistent expression of oncogenic TFs such as *MYC*, *TAL1*, and *MECOM* [14,15]. In medulloblastoma, structural rearrangements can reposition distal enhancers and activate *GFI1* and *GFI1B* [16]. Pan-cancer analyses identify recurrent enhancer hijacking at *IRS4* and *IGF2* [17]. Prostate cancer undergoes androgen receptor-driven enhancer reprogramming that supports castration resistance, whereas ERα-positive breast cancer acquires alternative enhancer networks that promote endocrine resistance through redistribution of *FOXA1* and estrogen receptor binding [15,18]. Similarly, enhancer remodeling contributes to activation of *WNT* and *MYC* signaling in colorectal cancer [14]. Together with findings in other cancers, these observations indicate that altered enhancer regulation recurs as a mechanism of oncogenic transcriptional reprogramming [14,15].

In this review, we use “enhancer–promoter rewiring” as an operational term for cancer-associated changes in one or more linked regulatory features, including enhancer activity or chromatin state, physical contact with a promoter, target-promoter selection, and the transcriptional consequences of these relationships. These features should not be treated as interchangeable. Active enhancer-associated features, such as chromatin accessibility and H3K27ac, indicate regulatory potential but do not establish a functional enhancer–promoter interaction. Similarly, chromosome-conformation assays measure spatial contact or contact enrichment but do not, on their own, demonstrate that an enhancer causally regulates a particular gene. We therefore distinguish four complementary evidence layers: (i) enhancer state and transcription-factor occupancy; (ii) physical contact measured using methods such as Hi-C, Micro-C, Capture-C, HiChIP, or imaging; (iii) promoter engagement or transcriptional initiation; and (iv) changes in RNA or protein output and the associated cellular phenotype. Strong causal claims are best supported by perturbation of the enhancer, promoter, or relevant interaction machinery, followed by measurement of transcriptional and phenotypic effects. This framework provides a practical basis for interpreting the literature; it does not imply that every study measures all four layers. Moreover, chromatin contact frequency and transcription can change independently, depending on the genomic locus and cellular context [8,9,10,19,20,21,22].

Studies of epigenetic therapy suggest that oncogenic transcriptional circuits can sometimes be disrupted without directly targeting mutant oncogenes. Pharmacological approaches targeting BET bromodomain proteins, histone acetyltransferases, histone deacetylases, *EZH2*, mutant *IDH* enzymes, and chromatin-remodeling complexes are being investigated in different cancer contexts [15,23,24]. However, reduced oncogenic transcription after treatment does not by itself demonstrate that enhancer–promoter contacts have been disrupted. Advances in CRISPR-based epigenome editing, single-cell multi-omics, spatial genomics, high-resolution chromatin-conformation mapping, and computational prediction are enabling more precise interrogation of non-coding regulatory elements and their potential therapeutic relevance [23,24,25,26].

In this review, we discuss how enhancer–promoter communication is established and maintained within the 3D genome and examine the molecular mechanisms and drivers underlying epigenetic rewiring of enhancer–promoter interactions in cancer, including alterations in chromatin architecture, TF networks, epigenetic regulators, and genome topology. We also highlight how the alterations of these epigenetic processes contribute to cancer initiation, progression, metastasis, and therapeutic resistance. Finally, we discuss epigenetic therapeutic strategies and emerging genomic technologies that exploit aberrant enhancer–promoter interactions to advance precision oncology.

## 2. Molecular Basis of Enhancer–Promoter Communication

### 2.1. Regulatory Landscape of Enhancers and Promoters

Cancer is increasingly recognized as a disease of transcriptional dysregulation in which aberrant gene expression arises not only from genetic mutations but also from extensive remodeling of the non-coding regulatory genome. Beyond alterations in protein-coding sequences, changes within cis-regulatory elements—including enhancers, promoters, and other chromatin regulatory regions—play fundamental roles in establishing and maintaining oncogenic transcriptional programs [9,14]. These regulatory elements function cooperatively within the 3D genome to coordinate precise spatial and temporal control of gene expression through the integration of transcription factor binding, chromatin accessibility, and epigenetic modifications [27,28,29]. Consequently, disruption of enhancer and promoter function has emerged as a central mechanism underlying malignant transformation and tumor evolution.

Enhancers are distal cis-regulatory DNA elements that recruit lineage-specific transcription factors, pioneer factors, chromatin remodelers, and transcriptional coactivators to regulate gene expression. Unlike promoters, enhancers generally function independent of orientation and can influence transcription over distances ranging from several kilobases to more than one megabase [27,30]. Genome-wide studies have identified hundreds of thousands of putative enhancers in mammalian genomes, most of which exhibit remarkable tissue specificity and are activated only in defined developmental or environmental contexts [31]. This cell-type specificity enables enhancers to establish lineage-restricted transcriptional programs while preserving cellular identity. Active enhancers are characterized by accessible chromatin and enrichment of histone H3 lysine 4 monomethylation (H3K4me1) and histone H3 lysine 27 acetylation (H3K27ac), together with recruitment of *CBP*/*p300*, Mediator, *BRD4*, RNA polymerase II, and bidirectional transcription of enhancer RNAs (eRNAs) [19,32,33,34]. These features promote TF occupancy, maintain an accessible chromatin environment, and facilitate communication with target promoters. In contrast, inactive enhancers are associated with condensed chromatin and repressive histone modifications, reflecting their transcriptionally silent state. Enhancer activity is highly dynamic and exists along a continuum of inactive, primed, poised, and active states. Primed enhancers are enriched for H3K4me1 but lack H3K27ac, whereas poised enhancers frequently carry both H3K4me1 and the repressive mark H3K27me3, enabling rapid activation in response to developmental or environmental cues [32,35]. This epigenetic plasticity allows cells to respond efficiently to differentiation signals, inflammatory stimuli, and metabolic stress. However, cancer cells frequently exploit this flexibility by reactivating developmental enhancers, establishing lineage-inappropriate regulatory elements, or generating de novo enhancers that promote oncogenic transcriptional programs [14,15].

Genome-wide epigenomic profiling has established enhancer remodeling as a hallmark of cancer. Rather than simply amplifying existing transcriptional programs, malignant cells acquire novel enhancer landscapes that support proliferation, survival, immune evasion, metabolic adaptation, and therapeutic resistance [15,36]. Integrative analyses using ATAC-seq, ChIP-seq, and single-cell multi-omics have revealed differences in regulatory chromatin across tumor types and heterogeneity in chromatin accessibility and putative enhancer activity among malignant cells [36,37]. These observations indicate that enhancer remodeling actively contributes to tumor evolution rather than representing a secondary consequence of malignant transformation.

Acute myeloid leukemia (AML) provides one of the clearest examples of enhancer-mediated transcriptional reprogramming. Leukemia-associated fusion proteins such as *RUNX1*/*ETO* compete with *RUNX1* and reshape the binding of hematopoietic TFs such as *PU.1* and *C/EBPα*, establishing disease-specific regulatory landscapes that sustain self-renewal while suppressing normal differentiation [38,39]. Single-cell chromatin accessibility studies further demonstrate substantial enhancer heterogeneity within acute leukemias, revealing regulatory states that correspond to distinct developmental stages [37,40]. Similar enhancer reprogramming has subsequently been described across numerous solid tumors, highlighting enhancer plasticity as a common feature of cancer.

While enhancers establish the regulatory potential of the genome, promoters determine whether these regulatory signals are converted into productive transcription. Promoters are cis-regulatory regions located adjacent to transcription start sites (TSSs) that recruit the pre-initiation complex, including RNA polymerase II, general transcription factors, Mediator, and additional chromatin-associated cofactors required for transcription initiation [29,41]. Beyond serving as sites of transcription initiation, promoters integrate regulatory inputs that influence transcription initiation, promoter-proximal pausing, elongation, and transcriptional bursting. Promoters exhibit substantial structural diversity. Broad promoters are typically associated with CpG islands and housekeeping genes, displaying multiple transcription initiation sites and constitutively accessible chromatin. In contrast, focused promoters generally contain a single dominant transcription start site and regulate developmental or tissue-specific genes that require tightly controlled enhancer-dependent activation [29,41]. These architectural differences contribute to gene-specific responsiveness to regulatory signals and are critical determinants of transcriptional precision. The epigenetic landscape of promoters further modulates transcriptional competence. Active promoters are enriched for H3K4me3, histone acetylation, accessible chromatin, and promoter-proximally paused RNA polymerase II, whereas inactive promoters frequently acquire DNA methylation and repressive histone modifications such as H3K27me3 [29,42]. Aberrant promoter hypermethylation represents one of the earliest and most pervasive epigenetic alterations in cancer, leading to stable silencing of numerous tumor suppressor genes while reinforcing oncogenic transcriptional programs [2].

Recent functional genomic studies have fundamentally revised the classical view of enhancer–promoter regulation by demonstrating that enhancer activity alone is insufficient to drive transcription. Instead, productive gene expression depends on selective enhancer–promoter compatibility, whereby only specific promoters respond to particular enhancers, despite widespread chromatin proximity [26,28]. CRISPR-based perturbation studies further revealed that many enhancers regulate genes located hundreds of kilobases away while bypassing nearby promoters, indicating that regulatory specificity cannot be predicted solely from linear genomic distance [20,21,25]. These findings indicate that promoter architecture, transcription factor composition, chromatin accessibility, and epigenetic state collectively determine whether a potential enhancer–promoter relationship results in productive transcription. Thus, enhancers and promoters function as interconnected regulatory modules rather than independent cis-regulatory elements. Defining how these elements communicate across the three-dimensional genome is therefore essential for understanding how regulatory information is converted into precise transcriptional outputs in normal development and cancer.

### 2.2. Molecular Mechanisms Governing Enhancer–Promoter Interaction

While enhancers and promoters define the regulatory potential of the genome, productive gene expression depends on the precise molecular mechanisms that enable communication between these cis-regulatory elements. Enhancer–promoter interactions are now understood as highly dynamic, probabilistic, and context-dependent processes rather than constitutive physical contacts. Recent advances in chromosome conformation capture technologies, live-cell imaging, CRISPR-based perturbation screens, and single-cell multi-omics have demonstrated that transcriptional activation results from the coordinated assembly of TFs, coactivators, chromatin remodelers, and RNA polymerase II (Pol II), whose interactions are continuously regulated in space and time [9,22,43]. Consequently, enhancer function is determined not simply by chromatin proximity but by the formation of transcriptionally competent regulatory complexes that selectively activate compatible promoters.

The first step in enhancer activation involves the cooperative binding of lineage-specific TFs and pioneer transcription factors (PFs) that recognize regulatory DNA sequences and establish accessible chromatin environments. PFs, including *FOXA1*, *GATA3*, *PU.1*, *OCT4*, and *SOX2*, possess the unique ability to bind nucleosomal DNA and recruit ATP-dependent chromatin-remodeling complexes that expose transcription factor binding sites inaccessible within compact chromatin [7,44]. These factors subsequently facilitate recruitment of lineage-determining TFs, histone acetyltransferases such as *CBP*/*p300*, and additional coactivator complexes that deposit activating histone modifications, particularly H3K27ac, thereby converting poised regulatory elements into transcriptionally active enhancers [27,28]. The combinatorial nature of transcription factor binding ensures that enhancer activity remains highly cell-type-specific and responsive to developmental and environmental signals.

**Figure 1:**
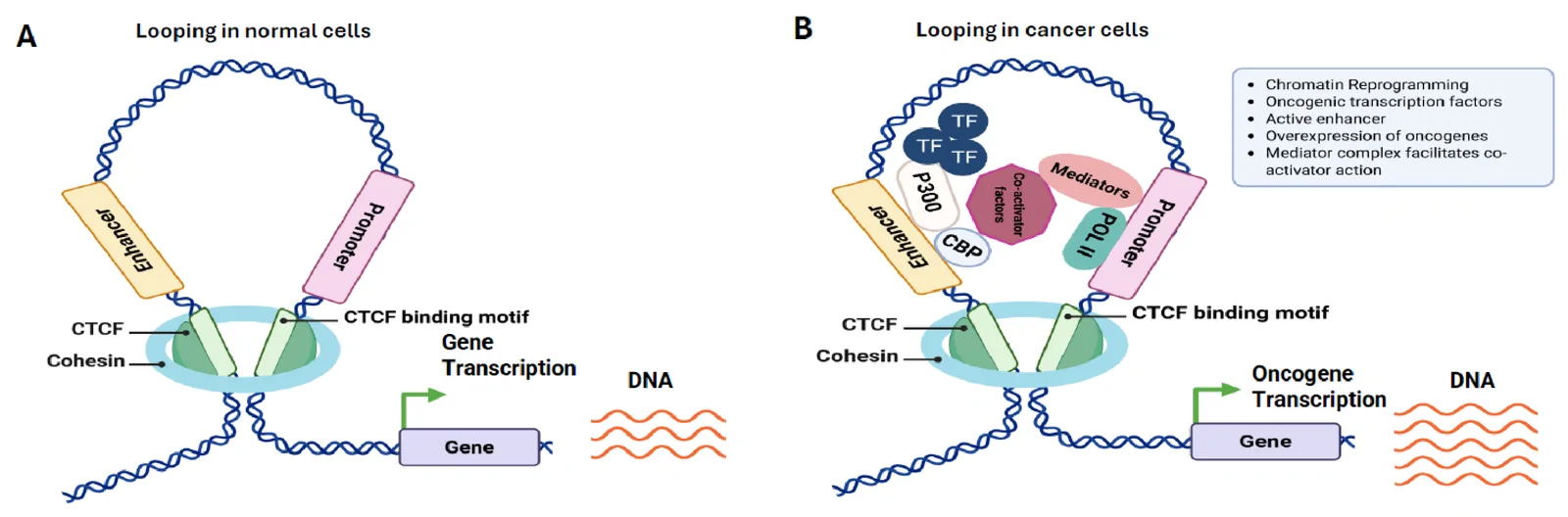
Enhancer–promoter looping and transcriptional activation in normal versus cancer cells. (**A**) Looping in normal cells: In this illustrative normal-cell model, CTCF proteins bound at convergent CTCF motifs, together with cohesin, help organize a chromatin loop that brings a distal enhancer into proximity with its target promoter. This architecture can support regulated enhancer–promoter communication and appropriate gene transcription. (**B**) Looping in cancer cells: One possible cancer-associated configuration is that enhancer activity and coactivator recruitment increase, including recruitment of oncogenic transcription factors, *CBP*/*p300*, Mediator, and RNA polymerase II, contributing to persistent expression of an oncogene. Cancer-associated rewiring is heterogeneous, however, and may also involve loss of contacts, altered promoter compatibility, or structural-variant-mediated enhancer hijacking; the specific mechanism must be established for each locus. In this illustrative model, the coactivator-dense chromatin state is depicted as accompanying persistent enhancer activity and elevated oncogene expression, in contrast to the tightly regulated transcription shown for normal cells. Created in BioRender. jalari, L. (2026) https://BioRender.com/bcdak6p.

A central component of enhancer–promoter communication is the Mediator complex, a conserved multiprotein coactivator that bridges enhancer-bound transcription factors with promoter-associated RNA polymerase II and the general transcription machinery. Genome-wide occupancy studies have shown that Mediator is preferentially enriched at active enhancers and super-enhancers, where it integrates multiple regulatory inputs to promote assembly of the pre-initiation complex and productive transcription initiation [33,45,46]. Loss of non-essential Mediator subunits primarily affects promoters linked to multiple enhancers, whereas Mediator itself is dispensable for physically tethering enhancers to promoters, indicating that it acts as a functional rather than an architectural bridge [47]. Mediator functions cooperatively with the cohesin complex, which supports enhancer–promoter communication by increasing contact frequency within regulatory domains. In this review, “stabilization” of an enhancer–promoter interaction refers to increased contact frequency or persistence in population-based measurements, rather than direct evidence of a stable physical complex. Although the detailed mechanisms of loop extrusion and higher-order genome organization are discussed in the following section, cohesin contributes directly to transcriptional regulation by increasing the frequency and duration of productive enhancer–promoter encounters. Functional studies indicate that cohesin depletion has modest effects on steady-state transcription but can impair inducible enhancer activity [48,49]. Importantly, recurrent mutations in cohesin subunits (*SMC1A*, *SMC3*, *RAD21*, and *STAG2*) occur in acute myeloid leukemia, where altered enhancer connectivity contributes to transcriptional dysregulation and impaired cellular differentiation (Figure 1) [50,51,52].

Enhancer–promoter communication may involve dynamic clustering of transcription factors, Mediator, *BRD4*, and other coactivators. Multivalent interactions among these proteins have motivated models in which transcriptional condensates concentrate regulatory machinery at active loci [53,54,55]. Such models may help explain aspects of super-enhancer organization, but the evidence that bona fide phase separation is necessary for enhancer function varies by system and remains under investigation. Condensates should therefore be discussed as a proposed mechanism in particular contexts, rather than as a universal explanation for enhancer–promoter communication.

Enhancer RNAs (eRNAs) are transcripts produced at many active enhancers and have been implicated in enhancer-mediated regulation [56,57]. Depending on the locus, eRNAs may influence cofactor recruitment, chromatin contacts, or target-gene expression. However, eRNA transcription, the RNA molecule itself, and the underlying enhancer DNA are experimentally distinct features. Their contributions should be separated using targeted perturbations where possible; eRNA detection alone does not establish that the transcript is required for enhancer function.

Recent CRISPR interference (CRISPRi) and CRISPR activation (CRISPRa) studies have fundamentally reshaped our understanding of enhancer–promoter specificity by demonstrating that physical proximity alone is insufficient to establish regulatory interactions. Instead, regulatory output depends on enhancer activity and contact frequency together with properties of the target promoter [20,21,25]. Massively parallel reporter assays that systematically pair thousands of enhancers and promoters further show that intrinsic enhancer–promoter compatibility ranges from broadly permissive to selective, shaped in part by promoter sequence and transcription factor motif content [58,59]. These findings help explain why many enhancers bypass the nearest gene to regulate more distant targets and why perturbation of individual regulatory factors can selectively alter transcriptional programs without globally disrupting chromatin architecture. Enhancer–promoter communication therefore emerges from coordinated interactions among transcription factors, coactivators, chromatin regulators, remodeling complexes, and architectural proteins. Together, these regulatory layers establish cell-type-specific transcriptional programs and provide the molecular framework through which enhancer–promoter networks can be remodeled during tumorigenesis.

### 2.3. From Chromatin Contact to Functional Enhancer–Promoter Regulation: Correlation Versus Causation

Enhancer chromatin state, physical enhancer–promoter contact, transcriptional initiation, and transcriptional output should be interpreted as distinct levels of regulation. Histone modifications such as H3K27ac, H3K4me1, and measures of chromatin accessibility help characterize enhancer state and activity, but do not independently establish contact with a particular promoter. Similarly, 3C, Hi-C, Micro-C, HiChIP, and related assays identify spatial associations between genomic regions; a detected or increased contact does not by itself prove that the interaction regulates the associated gene. Such contacts may be permissive for transcription, reflect a pre-existing regulatory relationship, or occur without a measurable transcriptional consequence. Conversely, changes in gene expression can occur without substantial changes in pairwise contact frequency [8,9,10,19,20,21,22].

Evidence should therefore be evaluated across complementary regulatory levels. Chromatin profiling can identify candidate active enhancers, conformation assays can identify candidate physical contacts, and measurements of RNA polymerase II engagement, nascent RNA, or steady-state RNA can establish whether these features are associated with transcriptional changes. However, combining observational assays does not automatically establish causality. Targeted perturbations—such as CRISPRi, CRISPR-mediated enhancer deletion, transcription-factor depletion, or disruption of a candidate interaction—provide stronger evidence when they produce the predicted change in target-gene expression. Appropriate controls and, where feasible, rescue experiments help distinguish direct regulatory effects from indirect consequences. Accordingly, “associated with” or “consistent with” is appropriate for observational evidence, whereas “drives,” “mediates,” or “is required for” should be reserved for relationships supported by functional evidence [20,21,45,47].

This distinction is especially important in cancer, where enhancer activation, transcription-factor redistribution, chromatin remodeling, altered contacts, and gene-expression changes may occur together during tumor progression or therapeutic resistance. Their coexistence does not establish the order of events or show that one change causes another. Structural-variant-mediated enhancer hijacking should also be distinguished from epigenetic activation of an existing enhancer: a genomic rearrangement can reposition an enhancer or create a new regulatory relationship, and the proposed target-gene effect should be supported by evidence appropriate to that mechanism. Explicitly distinguishing epigenomic association, measured chromatin contact, transcriptional consequences, and perturbation-supported causal dependence will make interpretations of enhancer–promoter rewiring more rigorous [14,16,17,60,61,62].

### 2.4. Molecular Drivers of Enhancer–Promoter Rewiring

The extensive remodeling of enhancer–promoter interactions described in the previous section reflects coordinated alterations in the molecular machinery that governs chromatin regulation and transcription. Rather than arising from individual genetic lesions alone, cancer-associated regulatory rewiring results from the combined activity of lineage-defining TFs, epigenetic modifiers, ATP-dependent chromatin remodeling complexes, and genome architectural proteins that collectively determine enhancer selection, promoter responsiveness, chromatin accessibility, and 3D genome organization. These regulatory systems function in a highly interconnected manner, whereby changes in one component frequently propagate through multiple layers of chromatin regulation to establish stable yet adaptable oncogenic transcriptional programs [7,9,27].

During tumor evolution, oncogenic signaling pathways and epigenetic alterations reshape these regulatory networks to generate cancer-specific enhancer landscapes, promote formation of novel enhancer–promoter interactions, and stabilize transcriptional dependencies that sustain proliferation, metastatic progression, immune evasion, and therapeutic resistance. Rather than functioning independently, transcription factors cooperate with chromatin modifiers, chromatin remodelers, Mediator, cohesin, and CTCF to establish dynamic regulatory hubs that integrate local chromatin modifications with higher-order genome architecture. Consequently, dysregulation of these molecular drivers represents a central mechanism through which cancer cells continuously remodel enhancer–promoter communication in response to developmental cues, microenvironmental signals, and therapeutic pressure [6,15,22]. The major molecular regulators and their contributions to cancer-associated enhancer rewiring are summarized in Table 1.

#### 2.4.1. TFs and Lineage-Specific Regulatory Networks

TFs are the primary determinants of enhancer selection and promoter specificity, serving as the molecular interface between extracellular signaling pathways and gene regulatory networks. By recognizing specific DNA motifs within enhancers and promoters, TFs recruit chromatin modifiers, coactivators, and components of the basal transcriptional machinery to establish cell-type-specific transcriptional programs. Among these regulators, PFs possess the unique ability to bind nucleosomal DNA within compact chromatin, initiating chromatin opening and creating accessible regulatory elements that subsequently recruit additional TFs and coactivator complexes [7,44,64]. Through this process, PFs establish enhancer repertoires that define lineage identity during development and maintain tissue-specific transcriptional programs in differentiated cells. In cancer, these regulatory mechanisms are extensively reprogrammed. Rather than simply altering transcription of individual genes, oncogenic transcription factors redistribute enhancer occupancy across the genome, reconfiguring enhancer programs and, in some contexts, altering enhancer–promoter contacts. This enhancer reprogramming is frequently driven by altered expression, mutation, amplification, or post-translational modification of lineage-defining transcription factors, resulting in widespread remodeling of chromatin accessibility and transcriptional circuitry [14,15]. Consequently, transcription factors function not only as downstream effectors of oncogenic signaling but also as master regulators of enhancer–promoter rewiring.

*FOXA1* represents one of the best-characterized pioneer factors involved in enhancer remodeling. In estrogen receptor-positive breast cancer, *FOXA1* cooperates with estrogen receptor α (ERα) to establish enhancer landscapes that regulate hormone-responsive transcription [77]. During acquisition of endocrine resistance, however, *FOXA1* undergoes extensive genomic redistribution, resulting in activation of alternative enhancer networks controlling cell-cycle progression, growth factor signaling, and epithelial plasticity [18]. Similar enhancer reprogramming occurs in prostate cancer, where androgen receptor (AR), *FOXA1*, and *HOXB13* cooperatively remodel enhancer landscapes during progression to castration-resistant disease, enabling persistent AR signaling despite androgen deprivation therapy [63,78].

Beyond hormone-dependent malignancies, oncogenic TFs including *MYC*, *AP-1*, *NF-κB*, *RUNX1*, *NOTCH1*, *TP63*, and *YAP*/*TEAD* reshape enhancer architecture across numerous cancers. *MYC* amplifies transcription from active enhancers and super-enhancers to support rapid proliferation and metabolic reprogramming, whereas *AP-1* and *NF-κB* establish inflammatory enhancer networks that promote invasion, immune evasion, and therapeutic resistance [15]. Likewise, *YAP*/*TAZ* and *TEAD* bind distal enhancers together with *AP-1* and activate genes that promote tumor cell growth [79]. Together, these TFs create highly interconnected regulatory circuits that continuously reshape enhancer landscapes during tumor evolution.

Recent single-cell chromatin accessibility and multi-omic studies further demonstrate that transcription factor occupancy exhibits substantial heterogeneity within individual tumors [37]. Rather than existing as fixed regulatory programs, enhancer networks dynamically respond to hypoxia, inflammation, metabolic stress, and therapeutic intervention, allowing cancer cells to transition between proliferative, invasive, stem-like, and drug-resistant phenotypes. This plasticity may help explain why enhancer reprogramming can occur rapidly in the absence of additional genetic mutations and highlights transcription factors as central mediators of epigenetic adaptation. Although transcription factors define enhancer identity and regulatory specificity, their regulatory activity depends on a broader epigenetic framework that governs chromatin accessibility and transcriptional competence. The establishment, maintenance, and interpretation of these chromatin states are orchestrated by epigenetic writers, erasers, and readers, whose coordinated activities determine whether enhancer–promoter communication is initiated, reinforced, or silenced.

#### 2.4.2. Epigenetic Writers, Erasers, and Readers Regulating Enhancer States

While TFs establish enhancer identity and recruit regulatory complexes, the functional state of enhancers is determined by dynamic epigenetic modifications that control chromatin accessibility, TF occupancy, and recruitment of transcriptional machinery. Epigenetic writers, erasers, and readers collectively regulate the transition between inactive, poised, and active enhancer states by depositing, removing, and interpreting chromatin modifications. During tumor progression, disruption of these regulatory systems results in persistent activation of oncogenic enhancers, inappropriate silencing of tumor suppressive regulatory elements, and establishment of cancer-specific enhancer–promoter networks [2,6].

Histone acetylation represents one of the most prominent mechanisms associated with active enhancer activation. Active enhancers are enriched for H3K27ac [19,32]. The histone acetyltransferases (HATs) *CBP* (CREBBP) and *p300* (EP300) acetylate H3K27, and inhibiting their catalytic activity reduces enhancer-associated acetylation and transcription [68]. In cancer, aberrant *CBP*/*p300* activity contributes to activation of oncogenic enhancer programs, particularly in tumors dependent on lineage-specific transcriptional networks. Opposing enhancer activation, histone deacetylases (HDACs) remove acetyl groups from histones and promote chromatin compaction, thereby limiting transcription factor accessibility and enhancer activity. Altered HDAC activity is frequently observed in cancer and contributes to abnormal silencing of differentiation-associated genes while maintaining transcriptional programs that support malignant phenotypes [2]. The balance between histone acetylation and deacetylation therefore represents an important determinant of enhancer accessibility and transcriptional competence.

Histone methylation provides another critical layer of enhancer regulation by defining enhancer competence and developmental state. The histone methyltransferases *MLL3* (KMT2C) and *MLL4* (KMT2D) are major enhancer-associated H3K4 mono-methyltransferases in mammalian cells and contribute to enhancer priming and activation in a context-dependent manner [69,70,71]. Loss of *MLL3*/*MLL4* function impairs enhancer activation and alters lineage-specific transcriptional programs [70,71]. Such findings suggest that enhancer dysfunction can arise not only through gain of oncogenic regulatory elements but also through loss of normal enhancer identity.

Conversely, repressive histone modifications contribute to enhancer silencing and maintenance of cellular identity. Polycomb Repressive Complex 2 (*PRC2*), through its catalytic component *EZH2*, deposits trimethylation on histone H3 lysine 27 (H3K27me3), maintaining developmental genes and regulatory elements in a repressed state [76]. Dysregulation of *PRC2* activity can alter the balance between active and repressed chromatin domains, contributing to inappropriate activation or silencing of regulatory programs during tumor evolution. At distal regulatory regions, transcription factor binding is associated with reduced DNA methylation [80]. The relationship is context dependent: methylation can accompany enhancer inactivity, while demethylation or TET-associated processes may facilitate activation at selected loci. Methylation should therefore be interpreted together with accessibility, histone marks, TF occupancy, and transcription rather than as a stand-alone readout of enhancer function.

Chromatin modifications are interpreted by specialized reader proteins that recognize specific histone marks and recruit additional regulatory complexes. Bromodomain-containing proteins such as *BRD4* recognize acetylated histones and facilitate recruitment of transcriptional machinery to active enhancers [72], whereas proteins recognizing repressive histone modifications promote maintenance of compact chromatin states. Through these coordinated writing, erasing, and reading activities, epigenetic regulators establish dynamic chromatin environments that determine enhancer activity and influence the probability of productive enhancer–promoter communication. The continuous deposition, removal, and interpretation of chromatin modifications enable enhancer activity to remain highly responsive to developmental, environmental, and oncogenic signals.

#### 2.4.3. Chromatin Remodeling Complexes Driving Enhancer Accessibility and Plasticity

The major ATP-dependent remodeling families include *SWI*/*SNF*, *CHD*, *ISWI*, and *INO80* complexes, each with distinct functions in chromatin organization. Mammalian *SWI*/*SNF* complexes include compositionally distinct *BAF*, *PBAF*, and non-canonical *BAF* (ncBAF) assemblies, which need not have identical genomic targets or effects. *SWI*/*SNF* components are frequently altered in cancer [73,74], and mutations in *ARID1A*, *SMARCA4*, *SMARCB1*, and *PBRM1* can alter nucleosome positioning, enhancer accessibility, transcription-factor binding, and lineage-specific gene-expression programs. The effects depend on which subunit or complex is altered and on the cellular context.

The role of *SWI*/*SNF* complexes extends beyond simple chromatin opening. These remodelers cooperate with transcription factors and coactivators to regulate chromatin accessibility and enhancer activity [73,74]. Thus, disruption of chromatin remodeling can alter not only enhancer accessibility but also regulatory communication between enhancers and their target promoters. Cancer cells can exploit chromatin remodeling mechanisms to generate transcriptional flexibility. Loss or mutation of remodeling complexes can create abnormal enhancer states that support tumor initiation, while adaptive remodeling enables established tumors to transition between transcriptional states during therapy. For example, altered *SWI*/*SNF* activity contributes to lineage plasticity in prostate cancer, allowing tumors to escape androgen receptor dependence and acquire aggressive neuroendocrine phenotypes [81]. Similarly, chromatin remodeling alterations promote stemness and therapy resistance by maintaining accessible regulatory landscapes associated with survival pathways.

Beyond regulating local chromatin accessibility, remodeling complexes influence the stability of long-range enhancer–promoter interactions through their functional interplay with architectural proteins that organize chromatin loops, topological domains, and nuclear compartments. These higher-order structures integrate local epigenetic states with global transcriptional regulation and help constrain enhancer–promoter communication.

#### 2.4.4. Architectural Proteins Shaping 3D Genome

3D genome organization provides the structural framework that constrains and facilitates enhancer–promoter communication. Architectural proteins establish chromatin loops, TADs, and higher-order nuclear compartments that determine which enhancers can communicate with specific promoters. In cancer, alterations in these regulatory systems can reshape chromatin architecture and are associated with aberrant enhancer–promoter interactions and oncogenic transcriptional programs; causal effects should be established at the relevant loci.

The CTCF–cohesin system is an important component of chromatin architecture and loop extrusion. Cohesin complexes, composed of *SMC1A*, *SMC3*, *RAD21*, and *STAG* proteins, extrude chromatin loops and are often constrained by convergently oriented CTCF-binding sites, contributing to insulated regulatory domains [11,66]. These structures can limit inappropriate enhancer activity, but they do not alone determine which enhancer activates which promoter. Disruption of CTCF binding or cohesin activity can alter insulation and selected chromatin contacts [48,82]; however, the transcriptional consequences vary by locus and cell context, and many enhancer–promoter contacts persist after acute architectural perturbation [83]. For example, in mouse embryonic stem cells, most enhancer–promoter interactions and transcription were maintained after 3 h depletion of *CTCF*, *cohesin*, *WAPL*, or *YY1*; because such experiments are acute and performed in few cell types, they address the maintenance rather than the establishment of contacts and may not extrapolate to tumors [83]. Mutations in cohesin components, cohesin-loading factors such as *NIPBL*, or *CTCF* boundaries may contribute to altered regulatory contacts, neo-TAD formation, or enhancer hijacking, but these outcomes should be demonstrated rather than assumed for each locus [60,67].

Beyond *CTCF* and *cohesin*, additional architectural regulators including *YY1*, Mediator, Polycomb proteins, and nuclear lamina-associated factors contribute to enhancer–promoter specificity and genome compartmentalization. *YY1*, for example, has been shown to facilitate enhancer–promoter looping at developmentally regulated loci [84], although acute *YY1* depletion had minimal effects on enhancer–promoter loops in mouse embryonic stem cells [83]. Polycomb complexes, in turn, organize repressive chromatin interactions that maintain lineage-specific gene silencing [76]. These factors illustrate that genome architecture is not governed by a single structural pathway but emerges from coordinated interactions among architectural proteins, transcriptional regulators, and chromatin-modifying complexes. Disruption of chromatin architecture can alter enhancer–promoter communication and cause inappropriate gene activation, as demonstrated in cancer and developmental disease models [16,60].

## 3. Epigenetic Rewiring of Enhancer–Promoter Interactions in Cancer

**Figure 2:**
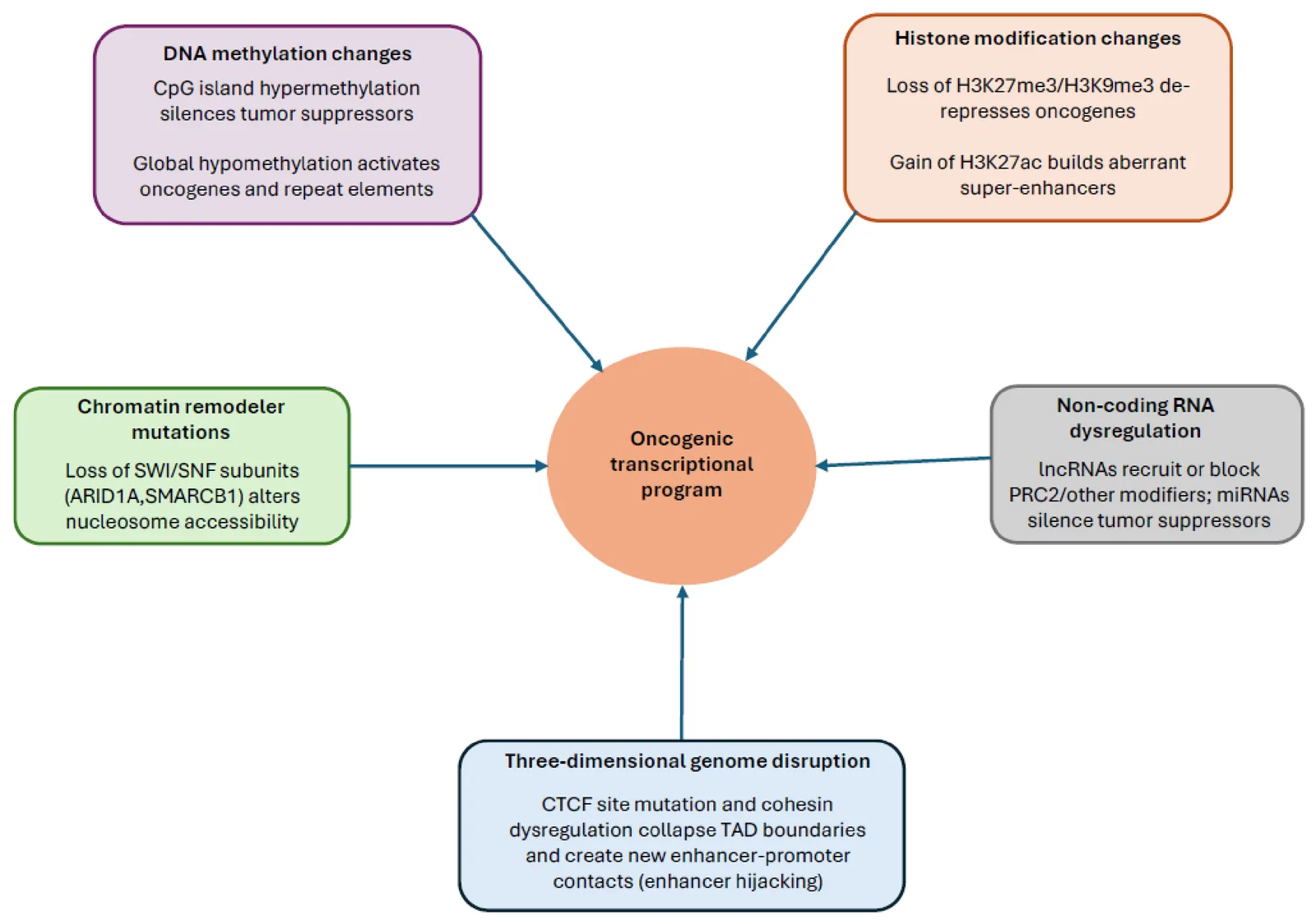
Epigenetic rewiring mechanisms in cancer. Cancer-associated alterations in DNA methylation, histone modifications, chromatin remodeling, non-coding RNA regulation, and three-dimensional (3D) genome organization converge to establish an oncogenic transcriptional program. DNA methylation changes include CpG island hypermethylation and global hypomethylation, which, depending on genomic context, can be associated with tumor-suppressor silencing or activation of oncogenes and repetitive elements. Histone modification changes, including loss of repressive marks (H3K27me3 and H3K9me3) and gain of active H3K27ac, can contribute to aberrant enhancer and super-enhancer activity in specific contexts. Mutations in chromatin remodelers, including *SWI*/*SNF* components such as *ARID1A* and *SMARCB1*, alter nucleosome accessibility and transcriptional regulation. Dysregulated non-coding RNAs, including lncRNAs and miRNAs, further modulate chromatin regulators and gene expression. Disruption of 3D genome architecture through CTCF-site mutations and cohesin dysregulation can weaken TAD boundaries and, at specific loci, permit aberrant enhancer–promoter interactions. Together, these interconnected mechanisms can remodel the cancer epigenome and 3D regulatory landscape and contribute to oncogenic transcriptional programs, tumor progression, and therapeutic resistance; their relative contributions vary by tumor type and locus.

The molecular mechanisms underlying enhancer–promoter interactions in normal cells are largely applied to cancer cells. During tumorigenesis, however, these regulatory systems are extensively reorganized, altering enhancer activity, TF occupancy, chromatin accessibility, and 3D genome architecture to support malignant cell states [14,15,36]. Rather than resulting solely from mutations in protein-coding genes, cancer-associated transcriptional dysregulation can arise from coordinated remodeling of noncoding regulatory elements and their interactions with target promoters. This regulatory plasticity allows cancer cells to establish new enhancer repertoires, reconfigure enhancer–promoter contacts, and redirect lineage-specific transcriptional programs toward proliferation, survival, invasion, metabolic adaptation, and immune modulation [6,37]. Because these regulatory states can change without requiring corresponding alterations in DNA sequence, enhancer–promoter networks provide a flexible substrate for tumor evolution and adaptation to changing cellular and microenvironmental conditions. The following sub-sections examine the major manifestations of this rewiring, focusing first on enhancer landscape reprogramming, followed by aberrant chromatin connectivity, super-enhancer reorganization, structural-variant-mediated enhancer hijacking, and epigenetic plasticity during tumor evolution. These interconnected mechanisms are summarized in Figure 2.

### 3.1. Reprogramming of Enhancer Landscapes in Cancer

One of the earliest and most pervasive epigenetic alterations during malignant transformation is the extensive remodeling of enhancer landscapes. Rather than simply amplifying pre-existing transcriptional programs, cancer cells establish novel enhancer repertoires while simultaneously inactivating lineage-specific regulatory elements. This redistribution of enhancer activity generates transcriptional networks that support proliferation, survival, metabolic adaptation, stemness, invasion, and metastatic progression [15,36]. Because enhancer states remain responsive to developmental and environmental signals, their reprogramming provides cancer cells with a flexible mechanism for adapting transcriptional output during tumor progression. Importantly, this remodeling may be sequence-driven, epigenetically mediated, or produced by cooperation between these mechanisms. Genetic events—including point mutations that create transcription-factor-binding motifs, copy number changes, and structural variants—can initiate or constrain a new regulatory configuration, whereas epigenetic adaptation can occur without a new DNA-sequence alteration through changes in transcription-factor abundance or occupancy, histone modifications, DNA methylation, nucleosome remodeling, or cell-state transitions. During tumor evolution, a genetic event may create a regulatory opportunity while epigenetic plasticity determines whether and when that opportunity is used. Accordingly, “epigenetic rewiring” in this review refers to changes in regulatory chromatin state and function and does not imply that every initiating event is non-genetic [6,14,17,18,60,61,62,78,85].

Cancer-associated enhancer remodeling reflects coordinated gains and losses of regulatory activity. Newly activated enhancers acquire features of transcriptionally permissive chromatin, whereas silencing of lineage-associated regulatory elements can disrupt differentiation programs and tumor-suppressive gene expression [7,27]. These changes are frequently associated with altered activity of lineage-defining transcription factors and oncogenic signaling pathways. Factors such as *FOXA1*, *GATA3*, *PU.1*, and *SOX2* contribute to cancer-specific enhancer repertoires, while oncogenic regulators including *MYC*, *AP-1*, *TP63*, *β-catenin*/*TCF*, and *YAP*/*TEAD* can reinforce enhancer programs associated with proliferation, stemness, metabolic adaptation, and invasion [15,44,86]. Thus, cancer-specific enhancer landscapes often emerge through convergence of lineage-associated regulatory circuitry with oncogenic signaling rather than through the activity of a single transcription factor.

Hormone-dependent cancers provide particularly well-characterized examples of this process. In estrogen receptor-positive breast cancer, changes in *ERα* and *FOXA1* occupancy can redistribute enhancer activity and generate alternative regulatory networks during endocrine resistance [77]. These changes can redirect transcription toward programs associated with cell-cycle progression, growth factor signaling, and cellular plasticity. Similarly, in prostate cancer, cooperation among *AR*, *FOXA1*, and *HOXB13* contributes to the reorganization of enhancer landscapes during progression to castration-resistant disease, allowing androgen-responsive transcriptional programs to persist despite androgen deprivation [63,78]. These examples illustrate how cancer cells can redirect established lineage regulatory circuitry toward alternative cellular states without necessarily requiring complete loss of the underlying lineage identity.

Enhancer reprogramming is also evident across diverse malignancies, including acute myeloid leukemia, glioblastoma, colorectal cancer, pancreatic ductal adenocarcinoma, melanoma, and neuroblastoma. In these settings, altered enhancer activity contributes to transcriptional programs governing proliferation, differentiation, epithelial-to-mesenchymal transition, stemness, and metastatic behavior [6,36]. Single-cell epigenomic studies further demonstrate that enhancer activity can vary substantially among malignant cells within the same tumor, with distinct enhancer repertoires and transcription factor networks coexisting within individual tumors [37]. In AML, single-cell RNA sequencing combined with genotyping has identified hierarchical malignant cell states and subclonal structure associated with disease progression and immune interactions, providing complementary transcriptomic evidence of cellular heterogeneity rather than direct evidence of enhancer accessibility or physical chromatin contacts [87]. Such heterogeneity provides a reservoir of regulatory states that can support phenotypic plasticity and facilitate tumor adaptation.

Overall, cancer-specific enhancer repertoires provide substantial transcriptional flexibility by altering which regulatory elements are active, which transcription factors occupy them, and which genes they can influence. However, enhancer activation alone does not determine transcriptional output. The functional consequences of enhancer remodeling depend on productive communication with target promoters within the three-dimensional genome, making altered enhancer–promoter connectivity a critical determinant of cancer-associated gene regulation.

### 3.2. Aberrant Enhancer–Promoter Looping and Chromatin Architecture

While enhancer reprogramming establishes the regulatory potential of cancer cells, its transcriptional consequences depend on the ability of enhancers to engage target promoters within the 3D genome. During tumorigenesis, genetic alterations, epigenetic remodeling, and changes in architectural proteins disrupt these constraints, producing aberrant enhancer–promoter communication that activates oncogenic programs and suppresses differentiation [9,22]. Cancer genomes can harbor de novo enhancer–promoter loops that are absent or substantially weaker in normal tissues; this has been shown most directly for neo-loops created by structural rearrangements [88], whereas for non-genetic changes, newly activated regulatory interactions should be distinguished from physically new loops. These long-range contacts can connect active enhancers with genes controlling proliferation, stemness, metabolism, immune evasion, and metastasis [13]. Conversely, loss or weakening of physiological enhancer–promoter contacts can reduce expression of lineage-specific genes and tumor suppressive programs, impair differentiation, and promote alternative cellular states [6]. Cancer-associated chromatin remodeling therefore involves both the formation of new regulatory contacts and the disruption of established interactions, collectively altering the transcriptional circuitry of malignant cells.

Architectural proteins provide an important regulatory layer in this process. Altered activity of cohesin components, including *STAG2*, *RAD21*, *SMC1A*, and *SMC3*, together with changes in *CTCF* occupancy, can modify chromatin insulation and loop organization [50,51,52,82]. Importantly, perturbation of cohesin does not necessarily result in a global collapse of genome architecture. Instead, specific regulatory loci can exhibit altered enhancer–promoter communication and transcriptional output, highlighting the context-dependent relationship between chromatin topology and gene regulation [48,83]. These observations indicate that cancer-associated architectural changes can selectively affect regulatory circuits rather than uniformly disrupting 3D genome organization.

Chromatin rewiring, including changes in enhancer activity, TF occupancy, chromatin accessibility, and epigenetic state, can alter the formation or stability of regulatory contacts and thereby reshape enhancer–promoter connectivity. In this context, lineage-associated and oncogenic factors such as *FOXA1*, *GATA3*, *MYC*, *AP-1*, and *YAP*/*TEAD* can cooperate with Mediator, cohesin, and chromatin-remodeling complexes to establish alternative regulatory configurations [15]. Such coordination provides a mechanism through which changes in local enhancer activity can be coupled to broader alterations in chromatin topology. Hormone-dependent cancers provide well-characterized examples of this dynamic relationship between TF redistribution and genome architecture. In *ERα*-positive breast cancer, altered *ERα* and *FOXA1* occupancy is associated with enhancer reprogramming and activation of alternative transcriptional programs [18,77]. Similarly, androgen receptor-associated chromatin organization is extensively remodeled during prostate cancer progression, generating alternative enhancer programs that maintain androgen-responsive transcription in advanced disease [63,78]. These examples demonstrate how changes in lineage-associated transcription factor activity can be accompanied by broader remodeling of regulatory topology.

Structural variants provide a distinct mechanism for disrupting these architectural constraints by altering genomic neighborhoods and repositioning regulatory elements. Deletions, inversions, duplications, and translocations can disrupt TAD boundaries or place enhancers in new regulatory contexts, enabling inappropriate enhancer–promoter interactions and oncogene activation [17,60]. Advances in Hi-C, Micro-C, HiChIP, PLAC-seq, and single-cell chromatin conformation profiling have further revealed substantial heterogeneity in enhancer–promoter connectivity within tumors [13,22]. Rather than representing fixed structural features, chromatin interactions can vary between individual cells and change in response to genomic instability, metabolic conditions, inflammatory signals, and other environmental cues. Altered chromatin topology therefore represents not merely a structural consequence of malignant transformation but an active mechanism through which cancer cells establish alternative regulatory relationships and generate diverse transcriptional states. One important consequence of this regulatory remodeling is the formation and reorganization of super-enhancers, which concentrate multiple enhancer activities and transcriptional cofactors at selected genomic regions. These regulatory domains can reinforce lineage-specific and oncogenic transcriptional programs and represent a distinct manifestation of cancer-associated enhancer reprogramming.

### 3.3. Super-Enhancer Reprogramming and Oncogenic Transcription

Super-enhancers (SEs) are operationally defined clusters of active regulatory elements that rank highly for features such as H3K27ac and often show substantial occupancy by lineage-defining transcription factors, Mediator, *BRD4*, *CBP*/*p300*, and other transcriptional cofactors [33,65]. Their identification depends on the assay and ranking procedure used, and the distinction between a super-enhancer and the upper end of a continuum of enhancer activity remains debated [89]. In many contexts, these clusters are associated with lineage- and state-defining genes and can integrate multiple regulatory inputs, but their designation alone does not prove a unique mechanism or exceptional functional dependence.

During malignant transformation, SE landscapes can undergo extensive reorganization. Cancer cells may acquire de novo SEs, expand existing enhancer clusters, or reactivate developmental SEs that are normally inactive in differentiated cells [65,90]. Because SEs integrate multiple regulatory elements within a single functional domain, alterations in transcription factor occupancy or cofactor recruitment across constituent enhancers can produce coordinated changes in the expression of associated genes. This organization enables cancer cells to reinforce lineage programs while incorporating oncogenic signals into transcriptional networks that support malignant cell states. Cancer-associated SE formation frequently reflects cooperation between lineage-defining and oncogenic transcription factors. Factors such as *FOXA1*, *GATA3*, *PU.1*, and *TP63* can cooperate with *MYC*, *AP-1*, *NOTCH1*, *β-catenin*/*TCF*, *NF-κB*, and *YAP*/*TEAD* to establish highly active enhancer clusters and recruit transcriptional coactivators [15]. Rather than functioning as isolated regulatory elements, these SE domains integrate multiple signaling pathways at selected genomic loci, thereby reinforcing transcriptional programs associated with proliferation, self-renewal, differentiation blockade, and other malignant phenotypes.

*MYC* provides a well-characterized example of SE-associated oncogenic transcription. Super-enhancer studies have shown that selected oncogenes can depend on concentrated enhancer activity and associated transcriptional machinery, although the degree of dependence varies by tumor type and locus [15,90]. Similar SE-associated regulatory programs have been described for *MYCN* in neuroblastoma [15]. These examples illustrate how SE reprogramming can consolidate regulatory activity around a restricted set of genes, whose sustained expression is important for malignant cell identity and tumor progression.

SE remodeling is also prominent in hematological malignancies. In AML, leukemogenic transcription factors and hematopoietic regulatory proteins establish leukemia-associated SEs that control programs involved in self-renewal, stemness, and differentiation [15,91]. In T-cell acute lymphoblastic leukemia, aberrant *NOTCH1* signaling contributes to the establishment of SEs associated with *MYC*, including a *NOTCH1*-controlled long-range *MYC* enhancer (N-Me) that lies within a broad super-enhancer region and is targeted by recurrent duplications [92]. Such changes illustrate how oncogenic signaling can reorganize developmental regulatory circuitry around transcriptional programs that sustain malignant cell states.

Importantly, SE activity is not uniform across all tumor cells. Single-cell epigenomic studies reveal heterogeneity in chromatin accessibility and regulatory programs among malignant cells, reflecting distinct cellular states within the same malignancy [37]. These differences can be associated with proliferative capacity, epithelial-to-mesenchymal transition, metastatic potential, immune interactions, and therapy-resistant states. SEs therefore represent dynamic regulatory configurations rather than fixed genomic entities, and their composition can change as tumor cells transition between phenotypic states or encounter different microenvironmental conditions.

Overall, SE reprogramming represents a particularly concentrated form of enhancer dysregulation in which multiple regulatory elements and transcriptional cofactors converge on genes that define malignant cell identity and state. Their dynamic organization enables cancer cells to reinforce oncogenic transcription while retaining the flexibility to adapt regulatory programs during tumor evolution. This distinctive combination of regulatory concentration and plasticity makes SE-associated transcriptional networks an important feature of cancer-specific enhancer reprogramming.

### 3.4. Enhancer Hijacking by Structural Variants

A major mechanism underlying cancer-associated regulatory rewiring is enhancer hijacking, in which structural alterations reposition enhancers into aberrant genomic contexts, enabling them to activate genes that are normally outside their regulatory neighborhoods. Unlike mutations that directly alter protein-coding sequences, enhancer hijacking can reprogram gene expression by changing the regulatory context of otherwise intact genes and creating novel enhancer–promoter relationships [17,60].

Several well-characterized examples demonstrate the oncogenic consequences of regulatory rewiring. In T-cell acute lymphoblastic leukemia, recurrent small insertions or deletions upstream of *TAL1* can create MYB-binding motifs and promote formation of an oncogenic super-enhancer, increasing *TAL1* expression; this mechanism should not be conflated with CTCF-boundary disruption unless direct evidence for altered insulation is shown in the specific study [93]. In medulloblastoma, structural rearrangements can reposition active enhancer clusters near *GFI1* or *GFI1B*, resulting in ectopic oncogene expression [16]. These examples illustrate distinct routes to oncogenic enhancer activation, including sequence-level creation of TF-binding sites and structural repositioning of regulatory elements.

Inversion of chromosome 3 [inv(3)] or t(3;3) in AML can relocate a distal *GATA2* enhancer toward the *EVI1*/*MECOM* locus, resulting in increased *EVI1* expression together with reduced *GATA2* expression [61]. This example illustrates how a single structural alteration can simultaneously rewire multiple regulatory relationships within a genomic region.

Structural enhancer rewiring can also activate *TERT*, the catalytic subunit of telomerase. Beyond recurrent promoter mutations, structural rearrangements and long-range enhancer repositioning can place *TERT* under the control of active regulatory elements in neuroblastoma [62,94]. These findings illustrate how distinct structural and regulatory mechanisms can converge on common oncogenic pathways by altering the regulatory environment surrounding an otherwise intact gene.

Structural variants can additionally generate new chromatin neighborhoods through formation of neo-TADs. Deletions, inversions, duplications, and other rearrangements can alter existing domain boundaries or create novel combinations of regulatory elements, bringing enhancers and promoters that were previously separated into the same regulatory domain [60]. In this context, enhancer hijacking represents not simply the movement of an individual enhancer but a broader reorganization of the regulatory landscape surrounding the affected locus. High-resolution chromosome conformation studies, including analyses that reconstruct rearranged cancer genomes, have provided direct evidence that structural variants can alter chromatin contacts and create candidate enhancer–promoter interactions [60,61,88].

Advances in long-read sequencing, optical genome mapping, and high-resolution chromosome conformation technologies have improved the detection and functional characterization of enhancer-hijacking events. Long-read sequencing and optical mapping can resolve complex structural variants that are difficult to reconstruct using short-read approaches, whereas Hi-C, Micro-C, HiChIP, PLAC-seq, and Capture-C can identify regulatory contacts associated with rearranged genomic regions [88,95,96]. Integration of these approaches with CRISPR-based functional genomics further enables investigators to determine whether a structural variant directly alters enhancer activity or enhancer–promoter communication rather than simply correlating with gene-expression changes.

Overall, enhancer hijacking demonstrates how structural variation can convert the three-dimensional genome into a source of oncogenic regulatory innovation. By relocating enhancers, altering genomic boundaries, or creating new regulatory neighborhoods, structural variants can establish aberrant enhancer–promoter relationships that activate oncogenes and disrupt normal lineage programs (Table 2). These newly established regulatory configurations can subsequently become integrated with transcription factor activity and epigenetic states, reinforcing malignant transcriptional programs during tumor progression.

### 3.5. Epigenetic Plasticity and Tumor Evolution

Epigenetic rewiring of enhancer–promoter networks is not a single event associated with malignant transformation but a continuous process that evolves as tumors acquire new cellular states. Cancer cells encounter hypoxia, nutrient limitation, inflammatory signals, stromal interactions, metabolic changes, and other microenvironmental cues that can alter enhancer activity, transcription factor occupancy, chromatin accessibility, and genome organization. Because these regulatory states can change without requiring alterations in the underlying DNA sequence, they provide a flexible mechanism for adapting transcriptional programs to changing cellular conditions [6,85].

Changes in chromatin accessibility, histone modifications, DNA methylation, enhancer activity, and 3D genome organization can therefore continuously reshape transcriptional programs during tumor evolution [36,37]. Single-cell multi-omics studies have revealed regulatory heterogeneity within tumors, including differences in chromatin accessibility, inferred enhancer activity, and transcriptional programs [37,99]. Integration of single-cell Hi-C and RNA-seq further enables investigation of chromatin organization in relation to transcriptional states [100]. These states can coexist within the same tumor and may dynamically interconvert in response to developmental signals, metabolic stress, inflammatory cues, or changes in the surrounding microenvironment. Such regulatory plasticity contributes to intratumoral heterogeneity by allowing genetically similar cells to occupy distinct transcriptional states. Cells may transition between proliferative, invasive, stem-like, differentiated, and metabolically distinct phenotypes through changes in enhancer activity and chromatin organization. These transitions can influence tumor growth, metastatic dissemination, immune interactions, and cellular survival under changing environmental conditions [6,37].

Hormone-dependent cancers provide clear examples of this regulatory plasticity. In ERα-positive breast cancer, changes in *ERα* and *FOXA1* occupancy can generate alternative enhancer networks and alter gene expression programs associated with endocrine resistance [18,77]. Similarly, progression of prostate cancer is accompanied by reorganization of androgen receptor-associated enhancers and super-enhancers, producing alternative regulatory states that maintain AR-dependent transcription during disease progression [63,78]. These examples illustrate how lineage-associated regulatory networks can be repeatedly reorganized as tumors progress and acquire new cellular states.

Epigenetic plasticity also shapes interactions between tumor cells and their microenvironment. Epigenetic therapy can restore antigen presentation and immune signaling in tumor cells [101]. Changes in enhancer accessibility and transcription factor occupancy can consequently alter inflammatory signaling, immune recognition, and the composition of the tumor microenvironment. These interactions can further reinforce distinct regulatory states within tumor cell populations and contribute to their adaptive capacity.

Overall, the dynamic nature of enhancer regulation allows cancer cells to continually reorganize transcriptional networks in response to changing cellular and environmental conditions. This plasticity emerges from coordinated interactions among transcription factors, epigenetic regulators, chromatin-remodeling complexes, coactivators, and genome architectural proteins, enabling genetically similar cells to occupy distinct regulatory states. Such adaptability provides a mechanistic basis for intratumoral heterogeneity, lineage plasticity, and tumor progression and establishes the regulatory context in which cancer-specific enhancer dependencies can emerge.

## 4. Therapeutic Targeting Enhancer–Promoter Interactions

The dynamic and reversible nature of enhancer–promoter rewiring in cancer creates opportunities to target regulatory dependencies that sustain tumor growth, survival, metabolic adaptation, and therapeutic resistance [15,36]. These dependencies arise through coordinated activity of epigenetic regulators, TFs, chromatin remodelers, transcriptional cofactors, and architectural proteins. Therapeutic strategies can therefore intervene at multiple levels of enhancer–promoter regulation, ranging from modulation of enhancer-associated chromatin states to disruption of transcriptional complexes and pathological chromatin contacts. Rather than broadly suppressing enhancer activity, an emerging goal is to selectively target regulatory dependencies that are preferentially acquired or reinforced by cancer cells. Evaluating these therapeutic strategies requires distinguishing changes in transcriptional activity from direct alterations in enhancer–promoter contacts. A drug-induced change in gene expression or enhancer-associated histone marks does not, by itself, demonstrate disruption of a physical enhancer–promoter interaction. Accordingly, the pharmacological effects discussed below are distinguished from direct evidence of altered chromatin contacts, which requires appropriate chromatin-conformation or imaging assays and, ideally, complementary perturbation experiments and functional validation [9,10,20,21,68,86,90].

### 4.1. Targeting Enhancer-Associated Epigenetic and Transcriptional Regulators

Enhancer activity is strongly influenced by dynamic chromatin states, making histone-modifying enzymes and chromatin readers important therapeutic targets. Active enhancers are generally characterized by accessible chromatin and enrichment of H3K27ac, which promotes recruitment of transcriptional cofactors and RNA polymerase II [2,6,15]. *CBP* and *p300* acetylate H3K27 at enhancers [68]. Aberrant *CBP*/*p300* activity can sustain oncogenic enhancer programs [102], whereas disruption of the *β-catenin*/*CBP* interaction altered 3D chromatin organization in pancreatic cancer cells [86], and the catalytic *CBP*/*p300* inhibitor A-485 reduces enhancer-associated acetylation and transcription [68]. HDACs provide an opposing mechanism by removing histone acetylation and influencing chromatin accessibility, although the effects of HDAC inhibition on enhancer activity are highly context dependent.

Histone methylation pathways provide an additional regulatory layer. *EZH2*, the catalytic component of *PRC2*, deposits H3K27me3 and represses developmental and differentiation-associated programs. Aberrant *EZH2* activity has been associated with malignant progression and therapy resistance, providing a rationale for targeting *EZH2*-dependent repression [76]. BET proteins, particularly *BRD4*, recognize acetylated chromatin at active enhancers and promote transcriptional elongation through interactions with Mediator and *P-TEFb* [72]. BET inhibition can therefore disrupt transcription from highly active oncogenic enhancers and super-enhancers [90].

Enhancer function also depends on transcriptional cofactors that connect regulatory DNA to productive transcription. Mediator facilitates communication between enhancer-bound transcription factors and RNA polymerase II, whereas *CDK7* and *CDK9* regulate transcription initiation and elongation, respectively [15,72,103]. Highly active oncogenic enhancers may exhibit increased dependence on these factors, creating opportunities to suppress transcriptional programs supported by strong enhancer activity. However, the effects of such interventions are unlikely to be uniform because enhancer strength, TF occupancy, and cofactor dependence vary among genes and tumor types.

### 4.2. Targeting Oncogenic Enhancer Networks and Chromatin Architecture

Super-enhancers represent concentrated regulatory domains enriched for TFs, cofactors, and transcriptional machinery and frequently control genes involved in lineage identity, proliferation, survival, and oncogenic signaling [33,65,90]. Their high dependence on transcriptional machinery can create therapeutic vulnerabilities. BRD4 inhibition, for example, can suppress super-enhancer-associated oncogenic transcription, including *MYC*-dependent programs in multiple myeloma [90]. These findings demonstrate transcriptional responses to treatment but do not establish that altered chromatin contacts mediate those responses. Similar dependencies have been described in neuroblastoma and other malignancies [15,103]. In ERα-positive breast cancer, *ERα* and *FOXA1* cooperate at enhancer networks controlling hormone-responsive transcription, whereas androgen receptor, *FOXA1*, and *HOXB13* establish lineage-specific enhancer programs in prostate cancer [63,77,78].

TFs therefore represent another important component of therapeutic targeting. *MYC*, *AP-1*, *NF-κB*, *YAP*/*TAZ–TEAD*, *β-catenin*/*TCF*, *NOTCH*, *FOXA1*, and *TP63* can establish or reinforce cancer-specific enhancer landscapes [15]. Advances in targeted protein degradation, structural biology, and protein–protein interaction inhibitors have expanded opportunities to interfere with previously difficult-to-target TFs and their regulatory partners. Nevertheless, because these factors control numerous genomic elements, therapeutic selectivity depends on differences in enhancer configuration and transcription factor dependence between malignant and normal cells.

Chromatin remodeling provides an additional level of intervention. *SWI*/*SNF*, *CHD*, *ISWI*, and *INO80* complexes regulate nucleosome positioning and accessibility of enhancer and promoter regions and are frequently altered in cancer [73,74]. Alterations in *SWI*/*SNF* components such as *ARID1A*, *SMARCA4*, *SMARCB1*, and *PBRM1* can reshape enhancer accessibility and transcriptional programs [74]. In some contexts, loss of one remodeling component creates dependence on a compensatory complex, providing opportunities for synthetic-lethal strategies. For example, *ARID1A*-mutant cancers depend on the paralog *ARID1B* [104].

The 3D genome further determines whether regulatory elements can communicate with their target promoters. *CTCF* and *cohesin* contribute to chromatin loop and TAD organization, whereas Mediator acts as a transcriptional coactivator that can influence regulatory communication [11,47]. Structural variants can disrupt domain boundaries and redirect enhancer activity toward inappropriate target genes [60]. Direct pharmacological targeting of chromatin architecture remains less developed than targeting epigenetic enzymes or transcriptional cofactors. However, tumors with altered cohesin or CTCF function may acquire specific architectural dependencies, while structural rearrangements that reposition enhancers near oncogenes can create highly selective regulatory vulnerabilities. CRISPRi, CRISPRa, dCas9-based epigenome editing, and programmable chromatin-loop perturbation provide complementary approaches for identifying and experimentally disrupting functional enhancer–promoter interactions. Importantly, detection of chromatin contact alone does not establish functional dependence, emphasizing the need to integrate chromatin-contact maps with enhancer activity, chromatin accessibility, transcription factor occupancy, and gene expression.

### 4.3. Combination Strategies and Biomarker-Guided Targeting

The plasticity of enhancer networks can limit responses to single-agent therapies. Cancer cells may activate alternative enhancers, redistribute transcription factors, or engage parallel regulatory pathways following disruption of a dominant regulatory program [85]. Combination strategies that target complementary regulatory layers may therefore provide more durable suppression. For example, simultaneous targeting of an enhancer-associated cofactor and its cooperating transcription factor could interfere with both enhancer activity and transcriptional output, whereas combined inhibition of chromatin-modifying enzymes and transcriptional elongation machinery may limit compensatory restoration of gene expression.

An important consideration is the distinction between broad epigenetic effects and tumor-specific regulatory dependencies. Global inhibition of chromatin regulators can affect normal tissues, whereas targeting a tumor-specific enhancer, transcription factor, or structural rearrangement may provide greater selectivity. Single-cell and spatial approaches can further identify distinct regulatory states within tumors and reveal subpopulations capable of compensatory enhancer rewiring. The heterogeneity of enhancer–promoter regulation also makes biomarker-guided treatment essential. Candidate biomarkers, which currently remain investigational or hypothetical in this setting, include enhancer and super-enhancer activity, TF occupancy, chromatin accessibility, histone modifications, structural variants, and recurrent enhancer–promoter contacts. Integration of these features with RNA-seq and single-cell multi-omics may identify regulatory dependencies that distinguish malignant from normal cellular states. Functional validation using CRISPR-based enhancer perturbation, chromatin-conformation assays, and transcriptional profiling can further distinguish causal dependencies from passive chromatin associations.

Overall, therapeutic targeting of enhancer–promoter interactions is moving from broad manipulation of epigenetic states toward more precise disruption of cancer-specific regulatory dependencies. Integrating enhancer activity, TF networks, epigenetic states, and 3D genome architecture may enable selective targeting of malignant regulatory circuits while preserving essential enhancer–promoter programs in normal tissues.

## 5. 3D Genomic Mapping Assays and Computational Methods for Enhancer–Promoter Interactions

3D genomic mapping assays provide complementary approaches for defining the spatial organization of chromatin and identifying candidate enhancer–promoter interactions (Table 3). Hi-C provides an unbiased, genome-wide map of chromatin contacts and is widely used to characterize compartments, TADs, and chromatin loops across the genome [11,105]. Micro-C extends this principle by using micrococcal nuclease digestion to achieve substantially higher spatial resolution, enabling detection of finer-scale chromatin contacts and interactions at near-nucleosome resolution [106,107,108]. In contrast, targeted approaches such as Capture-C, including NG Capture-C and Tiled-C, enrich predefined genomic regions before sequencing and therefore provide greater sensitivity for characterizing interactions involving selected promoters, enhancers, or regulatory loci [109,110,111]. Tri-C further enables examination of multiway interactions involving three or more genomic regions, providing information that can be obscured when higher-order contacts are reduced to conventional pairwise interactions [112]. Collectively, these approaches differ in genomic coverage, resolution, sensitivity, and ability to detect higher-order interactions, and their selection depends on whether the objective is to characterize global genome architecture or interrogate specific regulatory regions.

Chromatin-conformation assays generate sparse, distance-dependent contact data with assay-specific biases, and cancer genomes add further complexity through copy number alterations, structural variants, and extrachromosomal DNA (ecDNA). Extracting biologically meaningful outcomes therefore requires more than loop calling. Reads must first be converted into contact maps. Identifying loops within these maps then requires evaluating contacts against appropriate background models. A statistically enriched contact is not by itself evidence of a functional regulatory interaction. A loop can connect two loci that carry no active regulatory signal, or it can connect a genuine enhancer to a gene that it does not actually regulate. Establishing that a candidate enhancer–promoter interaction is functionally meaningful therefore requires evidence beyond the loop call itself. The candidate enhancer and promoter should carry the expected regulatory signatures, including chromatin accessibility and active histone marks such as H3K27ac and H3K4me1 at the enhancer and H3K4me3 at the promoter. The target gene’s expression should track the enhancer’s activity across cell types or conditions, consistent with the enhancer driving that expression rather than merely sitting near it. Where feasible, functional perturbation of the candidate enhancer, using approaches such as CRISPRi, CRISPRa, or CRISPR-Cas9 deletion, provides stronger causal evidence by testing whether altering the enhancer affects target-gene expression. Integrating contact data with chromatin signatures, gene-expression patterns, and functional perturbation results therefore helps distinguish candidate physical interactions from functionally supported enhancer–promoter relationships.

Additional sources of variation should be considered when comparing tumors or conditions. Copy number alterations can inflate or reduce apparent contact counts; tumor purity and stromal/immune admixture can dilute cell-intrinsic interactions; and cell-state heterogeneity can make a bulk contact map represent several distinct regulatory programs [113,114]. These effects can be partially addressed through CNV-aware normalization [113] and by accounting for estimated tumor purity where appropriate, while contact differences in heterogeneous samples should be interpreted in the context of their cellular composition. Biological replicates and appropriate normalization, with sequencing depth matching where necessary, can help distinguish biological changes from technical variation. Structural rearrangements require analysis against the rearranged, rather than only the reference, genome [60,61,62,95,96,114]. Sequencing depth, restriction or nuclease digestion, antibody enrichment, normalization, and loop-calling thresholds also influence which contacts are detected [111,115,116,117,118,119,120,121,122,123,124,125,126]. Computational or AI-based predictions should therefore be benchmarked against orthogonal assays and, where possible, perturbation data, with attention to potential training-data leakage, cell-type transferability, and performance on rearranged cancer genomes; correcting for copy number or estimated tumor purity reduces, but does not necessarily eliminate, these biases in highly rearranged or heterogeneous tumors [20,21,113]. Independent CRISPRi perturbation data can provide functional validation of predicted enhancer–gene relationships, while performance in primary tumors and rearranged cancer genomes requires separate evaluation.

Computational analysis of these data includes contact-map processing, statistical contact detection, multiway interaction analysis, enhancer–gene assignment, and reconstruction of rearranged cancer genomes (Table 4). For Hi-C and related proximity-ligation assays, pipelines such as HiC-Pro and Juicer perform alignment, filtering, and matrix generation, with ICE/KR balancing used to reduce coverage biases [115,116]. Micro-C is generally processed through the same read-to-contact-matrix workflow used for Hi-C, relying on pairtools and cooler [117,127]. Capture-C and its variants, including NG Capture-C, Tiled-C, and Tri-C, instead rely on dedicated pipelines such as CapCruncher, while CHiCAGO is widely used for interaction calling in Capture Hi-C data [111,118] (Table 4). Loop callers rely on different assumptions and are not interchangeable. Fit-Hi-C models distance-dependent background [120]. HiSIF uses a hidden Markov random field model to identify significant interactions [119]. HiCCUPS (implemented in Juicer) and Mustache detect focal enrichment using different strategies [11,121]. Peakachu applies supervised random forest classification to contact map features [122]. Assays enriched for a protein or histone mark require tools that account for this enrichment. HiChIP and PLAC-seq data are typically processed with hichipper, MAPS, or FitHiChIP, and ChIA-PET data with ChIA-PIPE [123,124,125,126]. ChIA-DropBox processes ChIA-Drop data, whereas MIA-Sig identifies statistically significant multiway chromatin interactions and has been applied to ChIA-Drop and SPRITE data. MATCHA uses hypergraph representation learning to analyze and predict multiway interactions from SPRITE and ChIA-Drop datasets. These methods retain information about multi-fragment chromatin complexes rather than treating them solely as independent pairwise interactions [128,129,130].

Physical proximity becomes informative when contact anchors are annotated with ATAC-seq or DNase-seq for accessibility, H3K27ac and H3K4me1 for enhancer state, H3K4me3 for active promoters, RNA-seq for transcriptional expression, and CUT&Tag as a low-input alternative for profiling chromatin binding proteins [131]. The Activity-by-Contact (ABC) model implements this same logic computationally, scoring candidate enhancer–promoter pairs from enhancer activity and contact frequency and validating predictions against large-scale CRISPRi perturbation data [20]. It is best understood as an enhancer–gene assignment framework, not a structural variant detector or a general loop caller.

Cancer genomes often carry deletions, inversions, translocations, and amplifications that can disrupt domain boundaries or reposition an enhancer near a different gene, a process termed enhancer hijacking. Hi-C maps from such genomes reflect the rearranged tumor chromosomes rather than the reference genome. NeoLoopFinder uses known SV breakpoints to reconstruct rearranged regions and re-examines Hi-C contacts in that context to identify neo-loops and enhancer-hijacking events [88]. These predictions can be compared with chromatin accessibility, H3K27ac, and gene expression data to evaluate their potential regulatory consequences. Because NeoLoopFinder depends on accurate breakpoints, complex or repetitive rearrangements are increasingly identified using long-read sequencing with SV callers such as Sniffles2, or optical genome mapping, which can span repetitive regions that are difficult to resolve with short-read sequencing [95,96]. ecDNA is a related but separate phenomenon. It refers to circular pieces of amplified DNA that exist outside the normal chromosomes. These amplicons can carry their own enhancers and can also form regulatory contacts with genes on the normal chromosomes. Because ecDNA involves a different kind of genome alteration, its structure, copy number, enhancer content, and contact behavior are usually studied separately from chromosomal rearrangements [97,98].

## 6. Conclusions and Future Perspectives

The epigenetic rewiring of enhancer–promoter interactions represents a defining frontier in functional genomics and precision oncology. Enhancers and promoters rely on a complex interplay of DNA methylation, histone modifications and 3D chromatin architecture, to orchestrate precise spatiotemporal gene expression. Disruption of these components can produce aberrant gene expression that contributes to cancer initiation, progression, metastasis, and therapeutic resistance. Understanding these changes requires distinguishing the molecular features associated with enhancer activity from the physical interactions and functional consequences they may produce.

A central interpretive principle is that enhancer activity, physical proximity, transcriptional output, and phenotype are related but distinct measurements. Future studies should state explicitly whether a conclusion is based on epigenomic association, measured chromatin contact, or perturbation-supported causal dependence. Integrating these evidence types—while accounting for genetic alterations, copy number, cell composition, and tumor heterogeneity—will improve the reliability of proposed regulatory mechanisms and therapeutic targets [8,9,10,20,21,22,85,114].

As the field of epigenomics moves from observational multi-omics toward targeted epigenetic engineering, we highlight several future perspectives on how studies of enhancer–promoter rewiring may reshape basic research and therapeutics. Firstly, the massive scale of multi-omics data has accumulated exponentially, enabling artificial intelligence-based approaches designed to map and predict enhancer–promoter interactions. Advances in machine learning have enabled the design of synthetic enhancers with experimentally validated tissue-specific activity [132], although their use to regulate oncogenic transcription remains exploratory. Secondly, future interventions may leverage programmable epigenome editors, which remain experimental. By using catalytically inactive Cas proteins (dCas9), or zinc fingers fused to epigenetic modifiers (such as DNMTs, TETs, HATs, or HDACs), it may be possible to selectively rewrite the epigenetic signatures of specific distal enhancers. For example, dCas9 fused to the p300 acetyltransferase core activated target genes from promoters and distal enhancers [133]. Lastly, future drug design strategies may focus on small molecules or small-molecule degraders capable of disrupting or stabilizing these multi-protein transcriptional hubs or biomolecular condensates, where these are shown to contribute to enhancer function.

A major hurdle in cancer therapy is epigenetic plasticity, where cancer cells bypass targeted therapies by rewiring their regulatory landscape. For instance, under therapeutic pressure, cancer cells can exhibit “promoter reinforcement”, a compensatory mechanism where enhancer–promoter interactions are lost, but alternative transcription factors relocate directly to the promoter to sustain oncogenic expression [134]. Therefore, the design of combination therapies to target these rewiring events might be a better strategy; simultaneously inhibiting primary active enhancers and blocking secondary promoter-reinforcement mechanisms could, in principle, limit the emergence of drug-resistant clones, although this hypothesis remains to be tested.

## Figures and Tables

**Table 1:** Molecular regulators of enhancer–promoter communication and their roles in cancer-associated transcriptional rewiring.

Regulatory Component	Examples	Function in Enhancer–Promoter Regulation	Contribution to Cancer-Associated Enhancer Rewiring	References
Lineage-defining transcription factors	*FOXA1*, *ERα*, *AR*, *GATA3*, *PU.1*, *MYC*, *YAP*/*TEAD*, *AP-1*	Establish cell-type-specific enhancer landscapes by binding regulatory elements and recruiting transcriptional cofactors	Redistribution of transcription factor binding creates tumor-specific enhancer programs that promote proliferation, lineage plasticity, and therapy resistance	[7,14,44,63]
Pioneer transcription factors	*FOXA1*, *GATA3*, *PU.1*, *OCT4*, *SOX2*, *TP63*	Recognize nucleosomal DNA and initiate chromatin opening, enabling recruitment of additional regulators	Reprogram enhancer accessibility during tumor progression and establish alternative transcriptional states	[44,64]
Mediator complex	*MED1*, *MED12*, *MED13*, *CDK8* module	Connects enhancer-bound transcription factors with promoter-bound RNA polymerase II and regulates transcription initiation	Enriched at oncogenic super-enhancers and associated with transcriptional addiction in cancer	[45,46,65]
Cohesin complex	*SMC1A*, *SMC3*, *RAD21*, *STAG1/2*	Drives loop extrusion and increases contact frequency between elements within regulatory domains	Mutations or altered cohesin activity can alter genome topology and gene regulation in a locus- and cell-context-dependent manner	[11,66,67]
CTCF architectural protein	*CTCF*	Establishes chromatin boundaries and insulates enhancer activity through loop formation	Loss of CTCF-mediated insulation can permit enhancer hijacking and inappropriate oncogene activation	[60,67]
Histone acetyltransferases (HATs)	*CBP*, *p300*	Deposit H3K27ac at active enhancers and recruit transcriptional machinery	Aberrant enhancer acetylation supports super-enhancer formation and oncogenic transcription	[19,68]
Histone methyltransferases	*KMT2C*/*MLL3*, *KMT2D*/*MLL4*, *EZH2*	Regulate enhancer priming, activation, and repression through histone methylation	Mutations alter enhancer states and promote malignant transformation and lineage instability	[2,69,70,71]
Histone demethylases	*KDM6A*/*UTX*, *KDM6B*	Remove repressive H3K27me3 marks to enable enhancer activation	Altered activity contributes to abnormal enhancer activation and tumor progression	[2]
Chromatin readers	*BRD4*, *BET* family proteins	Recognize acetylated histones and recruit Mediator/P-TEFb to activate transcription elongation	Maintain super-enhancer-driven oncogenic transcription and represent therapeutic vulnerabilities	[15,72]
Chromatin remodeling complexes	*SWI*/*SNF* (*BAF*), *CHD*, *INO80*, *ISWI*	Regulate nucleosome positioning and chromatin accessibility at enhancers and promoters	Mutations in ARID1A, SMARCA4, SMARCB1, and other components reshape enhancer landscapes	[73,74,75]
Enhancer RNAs (eRNAs)	Enhancer-derived noncoding RNAs	At selected loci, enhancer transcription or eRNA molecules are associated with cofactor recruitment, chromatin contacts, and target-gene regulation	Enhancer transcription has been linked to hormone receptor-dependent gene regulation	[56,57]
Polycomb complexes	*PRC1*, *PRC2*, *EZH2*	Establish repressive chromatin domains through H2AK119ub and H3K27me3	Promote silencing of differentiation genes and facilitate tumor cell plasticity	[76]

**Table 2:** Representative mechanisms of cancer-associated enhancer rewiring: cancer context, regulatory locus, target gene, initiating alteration, experimental evidence, and biological consequences.

Cancer Context; Regulatory Locus → Target Gene	Initiating Alteration (Genetic or Epigenetic)	Experimental Evidence and Biological Consequence	Interpretive Caution
ERα-positive breast cancer; *ERα*/*FOXA1*-bound enhancers → endocrine-response gene programs	Largely TF-driven: *FOXA1* upregulation and redistribution of *ERα*/*FOXA1* binding [18,77]	Differential ERα binding is associated with clinical outcome [77]; *FOXA1* upregulation promotes enhancer and transcriptional reprogramming in endocrine-resistant models [18]. Consequence: endocrine resistance.	Do not infer a changed physical contact from TF redistribution or RNA expression alone.
Prostate cancer; AR cistrome and an upstream AR enhancer → AR and AR-dependent programs	Epigenetic: reprogramming of the AR cistrome during tumorigenesis [63]. Genetic: somatic amplification of an upstream AR enhancer in advanced disease [78]	AR binding profiles in normal versus tumor tissue [63]; recurrent amplification of a noncoding AR enhancer identified as a driver in advanced prostate cancer [78]. Consequence: sustained AR signaling.	Changes in lineage programs do not necessarily imply that every associated loop is newly formed.
T-ALL; noncoding region upstream of TAL1 → TAL1	Genetic: somatic microinsertions that create *MYB*-binding motifs [93]	Patient sequencing, *MYB* and *CBP* recruitment, and formation of an oncogenic super-enhancer [93]. Consequence: monoallelic TAL1 overexpression.	Do not describe this as CTCF insulation loss unless the primary study directly demonstrates that mechanism.
T-ALL; *NOTCH1*-bound N-Me enhancer (+1.47 Mb) → MYC	Genetic: recurrent duplications of a *NOTCH1*-dependent enhancer [92]	Interaction with the *MYC* promoter; N-Me deletion in mice impairs thymocyte development and abrogates *NOTCH1*-induced T-ALL [92]. Consequence: *MYC*-driven leukemogenesis.	Enhancer activity is *NOTCH1*-dependent; consider sequence gain and TF-driven activity together.
Medulloblastoma; active enhancer clusters → *GFI1*/*GFI1B*	Genetic: structural variants that juxtapose *GFI1* or *GFI1B* with active enhancers [16]	Structural-variant mapping with chromatin-state and expression data supports enhancer hijacking [16]. Consequence: ectopic *GFI1*/*GFI1B* activation.	Reconstruct the rearranged genome when interpreting contact maps.
AML with inv(3)/t(3;3); distal *GATA2* enhancer → *MECOM*/*EVI1* (and *GATA2*)	Genetic: chromosomal rearrangement that repositions the enhancer [61]	Breakpoint mapping, chromatin-contact, and expression data [61]. Consequence: *EVI1* activation with reduced *GATA2* expression.	A single rearrangement can affect more than one gene; assess both gain and loss of regulatory activity.
AML; *BENC* enhancer cluster (~1.7 Mb downstream) → MYC	No sequence alteration required: activity of a pre-existing hematopoietic enhancer cluster [91]	Deletion in mice abolishes *Myc* expression in hematopoietic stem and progenitor cells; one module shows increased accessibility in human AML stem cells [91]. Consequence: *MYC* dosage in leukemic stem-cell hierarchies.	Example of enhancer-cluster dependency rather than a demonstrated rewiring event; accessibility differences in human AML are correlative, and dependency was shown mainly in mouse models.
Tumors with extrachromosomal DNA (ecDNA); ecDNA-borne enhancers → amplified oncogenes	Genetic: oncogene amplification on extrachromosomal circular DNA [97,98]	ecDNA can carry functional enhancers and act as mobile enhancers that amplify transcription [97,98]. Consequence: oncogene overexpression.	Treat ecDNA as distinct from a conventional chromosomal rearrangement.

**Table 3:** Comparison of representative assays and analysis approaches for enhancer–promoter studies.

Approach	Coverage/Resolution	Interaction Type	Input Requirements and Strengths	Main Limitations/Biases	Typical Role
Hi-C	Genome-wide. Resolution depends on sequencing depth and protocol	Pairwise	Requires crosslinked cells and deep sequencing for high resolution. Unbiased overview of compartments, domains, and candidate loops.	Contact maps are sparse and distance-dependent. High resolution can require substantial sequencing.	Survey global 3D architecture and compare conditions.
Micro-C	Genome-wide, often finer-scale than conventional Hi-C	Pairwise	Requires crosslinked cells, controlled MNase digestion, and deep sequencing. Nucleosome-scale fragmentation can resolve finer chromatin features.	Library preparation and sequencing depth affect sensitivity. Contacts remain associative.	Fine-scale loops and local genome organization.
Capture-C/Tiled-C	Targeted loci with high sensitivity	Pairwise	Requires predefined target regions and capture probes. Enrichment increases coverage at selected promoters or regulatory regions.	Requires prior target selection and does not provide an unbiased whole-genome view.	Focused testing of candidate regulatory contacts.
HiChIP/PLAC-seq	Genome-wide contacts enriched for a selected protein or histone mark	Pairwise	Requires a suitable antibody against the target protein or mark. Combines contact information with protein/mark enrichment.	Depends on antibody quality, target abundance, and assay-specific processing.	Map contacts associated with a regulator or chromatin state.
Tri-C/other multiway assays	Selected or genome-wide, protocol-dependent	Multiway	Requires assay-specific library preparation and, for Tri-C, predefined viewpoints. Can resolve interactions among three or more loci that pairwise assays may not fully represent.	Analysis is more specialized. Not directly interchangeable with pairwise loop calls.	Investigate higher-order regulatory hubs.
CRISPRi/CRISPRa and epigenome editing	Locus-specific functional perturbation	Not applicable	Requires guide RNA design, efficient delivery, and a gene expression readout. Tests whether an enhancer or chromatin feature contributes to target-gene expression.	Potential off-target effects and perturbation-specific artifacts. Contact change is not guaranteed.	Functional validation of candidate enhancer–gene relationships.
SV-aware analysis, long-read sequencing, optical genome mapping	Genome-wide or locus-specific structural-variant resolution	Not applicable	Requires high-molecular-weight DNA for long-read and optical mapping. Helps reconstruct rearranged cancer genomes and complex breakpoints.	Performance depends on sample purity, sequence complexity, and breakpoint accuracy.	Interpret enhancer hijacking, neo-TADs, and ecDNA-associated events.

**Table 4:** Computational tasks, representative tools, and supported assay inputs.

Task	Tools	Supported Assay Inputs
Contact-map processing	HiC-Pro [115], Juicer [116], pairtools [127], cooler [117]	Hi-C, Micro-C
	CapCruncher [111]	Capture-C, NG Capture-C, Tiled-C, Tri-C
Statistical contact detection	Fit-Hi-C [120], HiCCUPS [11], Mustache [121], HiSIF [119], Peakachu [122]	Hi-C and related contact matrices, including Micro-C where supported
	CHiCAGO [118]	Capture Hi-C (e.g., promoter capture Hi-C)
Contact processing and interaction detection	hichipper [123], MAPS [124], FitHiChIP [125]	HiChIP, PLAC-seq
	ChIA-PIPE [126]	ChIA-PET
Multiway interaction analysis	ChIA-DropBox [128], MATCHA [129], MIA-Sig [130]	ChIA-Drop and related multiplex interaction data, with compatibility depending on the tool
Enhancer–gene assignment	ABC [20]	Accessibility and H3K27ac with contact data
Rearranged-genome reconstruction	NeoLoopFinder [88]	Hi-C with SV breakpoints

## Data Availability

No new data were created or analyzed in this study. Data sharing is not applicable to this article.
